# Solitary Bees Acquire and Deposit Bacteria via Flowers: Testing the Environmental Transmission Hypothesis Using *Osmia lignaria*, *Phacelia tanacetifolia*, and *Apilactobacillus micheneri*

**DOI:** 10.1002/ece3.71138

**Published:** 2025-04-02

**Authors:** Magda Argueta‐Guzmán, Marko J. Spasojevic, Quinn S. McFrederick

**Affiliations:** ^1^ Department of Life & Environmental Sciences University of California Merced California USA; ^2^ Department of Entomology University of California Riverside California USA; ^3^ Department of Evolution, Ecology and Organismal Biology University of California Riverside California USA; ^4^ Environmental Dynamics and GeoEcology Institute University of California Riverside California USA

## Abstract

Microbial environmental transmission among individuals plays an important role in shaping the microbiomes of many species. Despite the importance of the microbiome for host fitness, empirical investigations on environmental transmission are scarce, particularly in systems where interactions across multiple trophic levels influence symbiotic dynamics. Here, we explore microbial transmission within insect microbiomes, focusing on solitary bees. Specifically, we investigate the environmental transmission hypothesis, which posits that solitary bees acquire and deposit their associated microbiota from and to their surroundings, especially flowers. Using experimental setups, we examine the transmission dynamics of *Apilactobacillus micheneri*, a fructophilic and acidophilic bacterium, between the solitary bee *Osmia lignaria* (Megachilidae) and the plant *Phacelia tanacetifolia* (Boraginaceae). Our results demonstrate that bees not only acquire bacteria from flowers but also deposit these microbes onto uninoculated flowers for other bees to acquire them, supporting a bidirectional microbial exchange. We therefore find empirical support for the environmental transmission hypothesis, and we discuss the multitrophic dependencies that facilitate microbial transmission between bees and flowers.

## Graphical abstract

We followed a sequence of flight cage experimental steps where we allowed bees to interact with bacteria‐inoculated flowers (10:30 am), before let them interact with new uninoculated flowers (11:30 am). Later, we allowed a new set of bees (1:30 pm) to interact with these shared flowers. At key experimental steps, we sampled bees and flowers to assess bacterial colony growth (curved arrows). Our results show that solitary bees can acquire and deposit bacteria from and onto flowers.

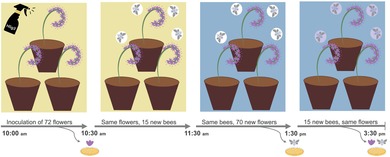

## 1 Introduction

Host‐associated microbiomes, like other biological communities, are assembled via four main processes: speciation, ecological drift, niche selection, and dispersal (Vellend 2010, 2016), with the distinction that niche selection (changes in species relative abundances resulting from deterministic fitness differences between species) and dispersal (the movement of species through space; Vellend 2010, 2016) inherently involve at least two trophic levels (e.g., the host's and the symbionts' trophic levels). Moreover, the strength of the association between the host and its microbiome members depends on the relative importance of dispersal mechanisms. For example, vertical transmission of microbes (from parents to offspring) promotes the evolution of heritable and consistent host–microbiome relationships (van Vliet and Doebeli 2019). In contrast, many hosts acquire their microbiomes from their environment (Douglas and Werren 2016), leading to more variable compositions in their microbiomes, at least at the taxonomic level. Thus, host dispersal is particularly important for shaping environmentally transmitted microbiomes, as host interactions with abiotic and biotic environments provide a potential platform for microbial acquisition.

Studies on microbial environmental transmission among pollinators have predominantly focused on social and managed species, such as honey bees ( *Apis mellifera* ) and bumblebees (*Bombus* spp.). For instance, bees and other pollinators interact with floral microbes on nectar and flower surfaces, facilitating microbial dispersal of both pathogenic and nonpathogenic microbes (Brysch‐Herzberg 2004; Ushio et al. 2015; Graystock et al. 2015; Adler et al. 2018; Russell et al. 2019; Figueroa et al. 2019). Floral nectar can selectively filter microbes introduced by pollinators (Herrera et al. 2008), and bumblebee foraging behavior has been shown to shape microbial dispersal patterns within floral communities (Kevan et al. 2007). These studies have significantly advanced our understanding of how microbes are transferred between social pollinators and their floral environments. However, microbial transmission between flowers and solitary bee species—representing the majority of bee diversity worldwide (Michener 2007)—remains largely untested in experimental studies.

For bacterial transmission to occur, two key processes are required: acquisition, where vector bees pick up microbes from source flowers, and deposition, where these microbes are transferred to new flowers. While studies on partially social bees, such as *Xylocopa appendiculata* , which can engage in communal nesting behavior, have demonstrated microbial deposition onto flowers (Kevan et al. 2007), the ability of solitary bees to acquire microbes from flowers and subsequently deposit them onto other flowers—thereby enabling microbial transfer to other solitary bees—remains untested. This knowledge gap underscores the need for research that examines both microbial acquisition and deposition in strictly solitary bee species.

The mutualism between bees and flowers is an important arena for environmental transmission of bacteria, where solitary bees not only may acquire microbes from the flowers they visit but may also deposit microbial communities back onto flowers (Adler et al. 2021). Solitary bees can transport microbes through two main routes: (1) externally via their exoskeleton and (2) internally via their gut microbiota. When bees visit flowers, they may pick up microbes on their body surfaces, particularly on the cuticle, as observed in *Osmia bicornis* (Megachilidae; Thamm et al. 2023) and in other flower‐associated insects such as butterflies, beetles, and thrips (Yamoah et al. 2008). Furthermore, some of these microbes may reach the bee digestive tract, potentially structuring the hindgut microbiome. Indeed, studies report the same bacterial strain in floral resources and in the bee–gut microbiota (McFrederick et al. 2017, 2018; Liu et al. 2023). This correlational evidence, showing similar microbial communities associated with the guts of multiple solitary bee species and the flowers they visit, strengthens the argument that bees, who emerge as adults with microbial blank slates (Koch and Schmid‐Hempel 2011; Martinson et al. 2012), acquire microbial symbionts through interactions with floral environments and other environmental sources. Furthermore, in a solitary ground‐nesting bee species ( *Nomia melanderi* ; Halictidae), newly emerged adults harbor microbial communities more similar to those found on nest walls than to those of older adults that have already foraged on flowers, possibly because their guts are empty at emergence time (Kapheim et al. 2021).

*Apilactobacillus micheneri* is a bacterium commonly associated with wild bees and flowers (McFrederick et al. 2017; Voulgari‐Kokota, McFrederick, et al. 2019). Known for its fructophilic nature, *A. micheneri* thrives in fructose‐rich environments, such as floral nectar and bee guts. Additionally, it possesses pectate lyase genes, which may play a role in the breakdown of pollen grains (Vuong and McFrederick 2019). However, the role of *Apilactobacillus* in bee health is still somewhat unclear. In some bee species, the pollen provisions of healthy larvae are dominated by *A. micheneri* (Hammer et al. 2023) but a lack of *A. micheneri* is not detrimental in other species (Brar et al. 2024). Although lactic acid bacteria are not a major part of the *Osmia lignaria* microbiome, the presence of these bacteria in the adult *O. lignaria* gut is linked to flower abundance (Cohen et al. 2020). In nature, *A. micheneri* has been isolated from the guts of various wild bee species, including *Caupolicana* (Colletidae), *Ptiloglossa* (Colletidae) (Hammer et al. 2023), *Nomia* (Halictidae) (Kapheim et al. 2021), *Megachile* (Megachilidae), and *Osmia* (Megachilidae) (McFrederick et al. 2017). Additionally, *A. micheneri* has been found in the pollen provisions from megachilids and colletids, as well as in floral environments visited by bees (Hammer et al. 2023; Voulgari‐Kokota, Ankenbrand, et al. 2019). Given that flowers and bees share *A. micheneri* (Lactobacillaceae) (McFrederick et al. 2017), we hypothesize that flowers are hubs of transmission for this bacterium among solitary bees. At a minimum, we predict that *A. micheneri* will be transferred via the bee exoskeleton to the floral environment, where the next visiting bee will acquire these bacteria. The flower environment as potential microbial transmission hubs incorporates an additional trophic level to consider in the dispersal dynamics of solitary bee microbiome assembly.

Here, we used an experimental approach to test the environmental transmission of *A. micheneri* among *Osmia lignaria* (Megachilidae) and *Phacelia tanacetifolia* (Boraginaceae). *A. micheneri* has been previously identified as a shared bacterial member in both flowers and solitary bee guts (McFrederick et al. 2017, 2018; Vuong and McFrederick 2019). This bacterium has also been found associated with the pollen provisions of ground‐nesting bees (Hammer et al. 2023), underscoring the narrow symbiotic relationship with solitary bees across their life cycle. Moreover, correlational evidence of environmental transmission of acidophilic bacteria suggests that flowers act as vectors of the bee microbiome components (McFrederick et al. 2012, 2017; Vuong and McFrederick 2019). *O. lignaria* , a solitary mason bee, is a generalist pollinator commonly used in agricultural pollination services. In lab settings, each *O. lignaria* individual emerges from its cocoon independently, making it an ideal solitary species for controlled experiments on microbial transmission. *P. tanacetifolia* , commonly known as lacy phacelia, is a widely used cover crop and pollinator‐friendly plant due to its abundant nectar and pollen resources and is commonly visited by *Osmia lignaria* in nature. We specifically ask the following *Does environmental transmission occur from flowers inoculated with A. micheneri to uninoculated solitary bees? If so, can these bees deposit the acquired bacterium onto uninoculated flowers where the bacteria can then be transmitted to other bees?*

## 2 Materials and Methods

### 2.1 Materials

We grew *Phacelia tanacetifolia* from seed in a greenhouse at the University of California Riverside (hereafter UCR) in Riverside, CA, USA, under conditions that excluded pollinators and insects, and we monitored the plants until they bloomed. We acquired *O. lignaria* cocoons from the Foothill Bee Ranch, Foresthill, California, and stored them at 4°C until the beginning of our experiment. For the bacterial component, we used *A. micheneri* strain HLIG3, which we had previously isolated from adult gusts of *Halictus ligatus* (Halictidae), a wild bee species (McFrederick et al. 2018), and had prepared glycerol stocks to preserve it. For this experiment, we cultured HLIG3 on MRS agar plates supplemented with 2% fructose, performing six successive subcultures to ensure the purity of the colonies (McFrederick et al. 2018). We selected *P. tanacetifolia* because, to the best of our knowledge, *A. micheneri* has not been reported on its flower surfaces in nature. This provided additional confidence that the presence of *A. micheneri* in our flowers resulted from our inoculation steps rather than external contamination.

### 2.2 Experimental Procedures

To test the environmental transmission of *A. micheneri* between flowers and bees and vice versa, we followed the protocols developed by Graystock et al. (2015). We conducted an experiment in April 2024 at the Entomology Department of UCR. We started by confirming that our *Osmia lignaria* bees and *Phacelia tanacetifolia* flowers were free of *Apilactobacillus micheneri*. Before each experimental step, we dissected the crop and hindgut of five randomly selected bees and sampled five whole flowers. To account for potential bacteria in the exoskeleton, we did not conduct surface sterilization on the bees before the aforementioned dissection. We used sterile phosphate‐buffered saline (PBS) to vortex our samples, and then we plated 100 μL of each sample on MRS agar plates supplemented with 2% fructose. After confirming no growth of *A. micheneri*, we proceeded with the rest of the experiment.

**FIGURE 1:**
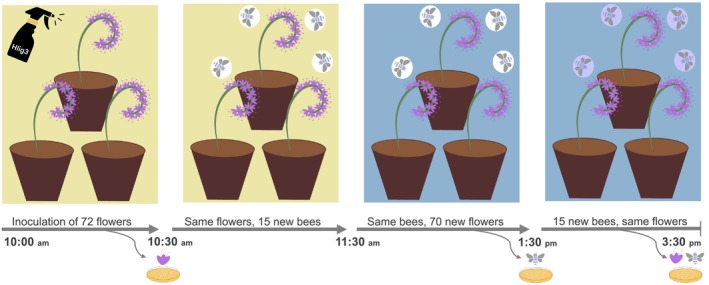
Experimental design to test the environmental transmission of *Apilactobacillus micheneri* between *Phacelia tanacetifolia* and *Osmia lignaria* . We sprayed 72 *P. tanacetifolia* flowers (pale yellow background) with *A. micheneri* (Hlig3) and allowed them to incubate for 30 min. Then, we introduced 15 newly emerged *O. lignaria* vector bees (white circles) to forage on these inoculated flowers for 1 h. After this period, we replaced the inoculated flowers with 70 new flowers (light blue background) and allowed the same vector bees to continue foraging for an additional 2 h. Finally, we removed the vector bees and introduced 15 new recipient bees (purple circles) to interact with the now shared flowers for 2 h. We marked the experimental steps after which we collected a minimum of 10 flowers and/or bees for plating to examine *A. micheneri* colony growth (thin gray curved lines).

We grew *A. micheneri* in 50 mL of MRS media supplemented with 2% fructose, to a bacterial concentration of approximately 10^5^ cells per μL. In bee guts, *A. micheneri* has been quantified up to a million cells in wild megachilids (same family than *O. lignaria* ; McFrederick et al. 2017). We incubated *O. lignaria* cocoons in a single flight cage at room temperature and under stable laboratory conditions. Following emergence, we subjected the individual bees to a 1‐day starvation period prior to the experiment. We sprayed 70 *P. tanacetifolia* flowers with the prepared liquid culture (hereafter referred to as inoculated flowers; Figure 1). To maximize coverage across different floral structures, we sprayed 800 μL of culture media onto the floral clusters, delivering approximately one million bacterial cells per flower. In nature, microbial cell densities (including bacteria and yeasts) on external floral structures such as petals and stamens range from 9000 to 1.5 million cells (Russell and Ashman 2019). Additionally, microbial densities in nectar can reach up to 10 million cells per μL (Rering et al. 2018). The following experimental steps took place outside under sunny or mostly sunny conditions (18°C–21°C), with a single trial in a single cage for each treatment (the transmission and control treatments). Thirty minutes after flower spraying, we placed the inoculated flowers into a new flight cage (100 × 100 × 152 cm), in which we placed 15 *O. lignaria* bees (hereafter vector bees) in a mesh flight cage, allowing them to forage for 1 h (Figure 1). Subsequently, we removed all inoculated flowers and introduced 72 non‐inoculated flowers (hereafter shared flowers) to the same cage. We allowed the vector bees to forage on these shared flowers for 2 h (Figure 1).

After the foraging period, we removed all vector bees and introduced 15 new bees (hereafter recipient bees), which had been exposed to neither *A. micheneri* nor the previously inoculated flowers. These recipient bees were allowed to interact with the 72 shared flowers for another 2 h (Figure 1). To control for bacterial transmission mechanisms and exclude airborne transmission or contamination, we established a control flight cage setup that we ran simultaneously under the same environmental conditions. Within this cage, 15 non‐inoculated bees were allowed to interact with 78 non‐inoculated flowers, mirroring the procedural steps observed in the experimental steps but solely with non‐inoculated participants (Figure 1). During all experimental steps, we observed that bees spent most of their time actively foraging on the flowers. At the end of the experimental steps, we sampled 10 individuals from each group: vector bees, inoculated flowers, shared flowers, control flowers, and control bees, except for recipient bees, from which we sampled 12 individuals. To remove, introduce, and collect bees and flowers between experimental steps, we carefully transported the cage to a dark room with red light in the McFrederick Lab at UCR to prevent the bees from escaping.

Under sterile conditions, we dissected the bee guts, including the crop to the hindgut. We deliberately chose not to perform surface sterilization on the bees before dissection. This decision was made to preserve the integrity of the microbial communities potentially associated with both the guts and the exoskeleton. To sample the flowers, we used sterile tweezers and grabbed the whole flower by its peduncle. Each sampled flower was placed in a sterile vial. For the preparation of bee gut samples and flowers, we utilized PBS to maintain isotonic conditions during the processing steps. We homogenized the bee gut samples using a sterile pestle in a microcentrifuge tube to ensure thorough breakdown of tissue. Concurrently, we subjected the flowers to vigorous shaking in PBS using a shaking incubator. We plated a 100 μL of these samples on a highly selective agar (MRS supplemented with 20% fructose) to assess the presence of *A. micheneri*. After 12 days of growth at room temperature, we counted *A. micheneri* colonies with a count detection maximum of 100 CFU. To confirm the taxonomic identity of the bacterial growth as *A. micheneri*, we extracted DNA from four bacterial colonies per experimental step (*n* = 16 colonies) using the Macherey‐Nagel (Duren, Germany) NucleoSpin Rapid Lyse Kit, adhering to the manufacturer's protocol for fresh samples. We then conducted PCR on the extracted DNA using the 27F and 1492R universal bacterial primers (Turner et al. 1999). We sent the samples to the Genomics Core at UC Riverside for Sanger sequencing. To verify the identity of the colonies as *A. micheneri HLIG3*, we performed BLAST searches of the sequenced DNA against the nr/nt database on NCBI.

### 2.3 Statistical Analyses

We recorded the frequency of flowers and bees on which *A. micheneri* was detected using highly selective agar plates (MRS supplemented with 20% fructose). To compare the presence of *A. micheneri* among experimental steps and controls, we fitted a logistic regression model with Firth's correction for likelihood in cases of data separation (i.e., all controls with 0 bacteria prevalence) and calculated the *χ* ^2^ statistic using the R package *logistf*. Finally, we compared the number of *A. micheneri* colonies per treatment using a Jonckheere–Terpstra test (Daniel 1990), which compares medians among groups when there is an expected order in the treatments.

## 3 Results

**FIGURE 2:**
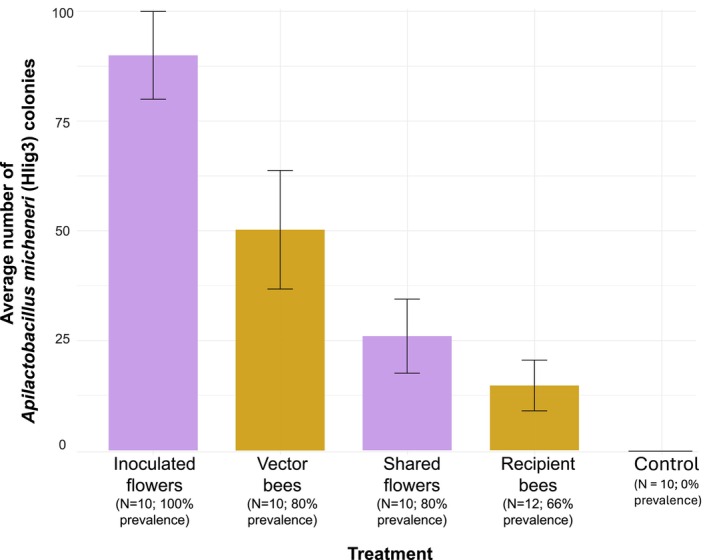
Average number of *A. micheneri* (Hlig3) colonies per experimental step in our single cage trial and the control cage. The bars represent SE values. We indicate prevalence of *A. micheneri* per experimental step.

We did not detect *A. micheneri* in the flowers or bees from the control treatment. During our experiment, we observed the bees actively foraging (i.e., collecting nectar and/or pollen) on the provided flowers. We detected *A. micheneri* in 90% of the sampled inoculated flowers (*χ* ^2^ = 22.36, df = 1, *p* < 0.01) and in 80% of the sampled vector bees (*χ* ^2^ = 17.48, df = 1, *p* < 0.01). Moreover, we detected *A. micheneri* in 80% of the sampled shared flowers (*χ* ^2^ = 13.9, df = 1, *p* < 0.01), found *A. micheneri* in 66% of the sampled recipient bees (*χ* ^2^ = 13.38, df = 1, *p* < 0.01), and observed a trend toward the average number of colonies per treatment decreasing after each experimental round (Figure 2; *J* = 270.5, *p* = 0.09). Finally, BLAST searches confirmed the identity of *A. micheneri* for all 16 of the recovered bacterial colonies in all experimental steps (minimum percent identity of 98%).

## 4 Discussion

Our study provides direct experimental evidence of microbial environmental transmission from flowers to solitary bees and vice versa. Previous studies have provided phylogenetic and correlational evidence suggesting that solitary bees and flowers share and exchange microbes, especially the bacterium *A. micheneri* (McFrederick et al. 2012, 2017, 2018; Vuong and McFrederick 2019). These studies have focused on analyzing the microbiota of flowers both isolated from and exposed to solitary bees, as well as the microbial communities in solitary bee guts and pollen provisions. By using inoculated flowers and monitoring bacterial transfer, we demonstrated that *A. micheneri* is spread among solitary bee individuals and between bees and flowers. Moreover, we found that solitary bees ( *O. lignaria* in this case) can acquire *A. micheneri* from inoculated flowers and then deposit it onto uninoculated flowers, where other bees can acquire the same bacteria. Our findings provide evidence that supports the environmental transmission hypothesis (McFrederick et al. 2017), which posits that interactions between bees and flowers create a network of microbial exchange within bee populations. While previous studies on social bees (honey bees and bumble bees) have demonstrated the transmission of eukaryotic parasites via flowers (Graystock et al. 2015; Figueroa et al. 2019) and nonpathogenic bacteria (Russell et al. 2019), our results suggest that solitary bees also move putatively beneficial bacteria from and onto flowers.

In our system of study, *A. micheneri* is thought to play a vital role in *Osmia* survival by potentially enhancing nutrient absorption or defending against pathogens when present in the gut microbiota and pollen provisions (Dharampal et al. 2019, 2022; Steffan et al. 2023). However, its absence in *Megachile rotundata—*a different solitary bee species—does not appear to negatively affect adult survival (Brar et al. 2024). The widespread occurrence of *A. micheneri* across different wild bee taxa (McFrederick et al. 2012, 2017; Voulgari‐Kokota, McFrederick, et al. 2019; Voulgari‐Kokota, Ankenbrand, et al. 2019; Kapheim et al. 2021; Hammer et al. 2023) suggests that its association with bees is both persistent and ecologically relevant, spanning different stages of the solitary bee life cycle. Similarly, in other invertebrate microbial symbioses, environmentally acquired microbes can provide essential benefits such as nutrient synthesis (as seen in the bean bug—*Burkholderia* system) and bioluminescence (as in the bobtail squid—*Aliivibrio fischeri* system), which are crucial for the host's survival (Kikuchi and Yumoto 2013; Nyholm and McFall‐Ngai 2021). In both systems, hosts acquire bacteria from abiotic sources like water and soil each generation. A distinctive feature of our system is the multitrophic dependency, where bees act as vectors, depositing bacteria onto flowers. These flowers, in turn, shape the microbial pool available to subsequent bee visitors. This two‐step process could play a pivotal role in structuring differences in the microbial diversity observed among bee species and wildflowers (Argueta‐Guzmán et al. 2024), enhancing our understanding of how microbial communities assemble in hosts that acquire their symbionts from the environment.

While we have empirically demonstrated the potential for environmental transmission between flowers and solitary bees, our results also motivate other lines of inquiry. For instance, most of the extant knowledge on how dispersal shapes microbial colonization of flowers focuses on priority effects (*sensu* Chase 2003), where the order and timing of species arrival influence community structure (Fukami 2015; Fukami et al. 2016). This historical contingency can have persistent impacts, influencing community dynamics across multiple generations (Toju et al. 2018). While the precise impact of the order in which bacteria arrive in the bee guts or on the exoskeleton remains unclear, the notable dominance of *A. micheneri* in the gut microbiota of various wild bee species as well as in pollen provisions (McFrederick et al. 2018; Hammer et al. 2023) may stem from priority effects driven by bee‐mediated dispersal and the variety of flowers visited.

Future studies could also explore how microbial transmission varies among different solitary bee species and how/if specific flowering plant species serve as prominent hubs for beneficial and commensal microbes. Such research could have significant implications for biodiversity conservation and agricultural practices by helping identify flower species that not only maximize nutritional value but also sustain the transmission of beneficial microbes. Moreover, climate change literature has highlighted the existence of phenological mismatches between flowering plants and their pollinators, as well as the potential consequences for their mutualistic interactions (Rafferty and Ives 2011). However, an emerging aspect to consider regarding the effects of plant extinction due to climate change is the potential loss of beneficial microbial sources for the bees. Therefore, further research should also focus on identifying the plant traits that best promote the transfer of beneficial microbes and test how a changing climate (e.g., heat waves) might affect the efficacy of environmental microbial transmission.

### 4.1 Study Limitations

While our experiment provides evidence for the environmental transmission of *A. micheneri* between flowers and solitary bees, two limitations should be considered when interpreting our findings. First, there is the potential for microbial transmission via the mesh cage itself. Although our experiment was designed to test floral transmission, we did not account for the possibility that bees could acquire *A. micheneri* from surfaces other than flowers, such as the mesh of the cage. If *A. micheneri* was deposited onto the mesh by bees that interacted with inoculated flowers and later picked up by new bees, this would still demonstrate that *A. micheneri* can be transmitted via environmental surfaces, reinforcing the broader concept that solitary bees can acquire bacteria from multiple environmental sources and subsequently deposit them elsewhere, including onto flowers. Future studies could mitigate this concern by incorporating additional controls, such as cages where no flowers are introduced, to determine whether transmission occurs in the absence of floral contact.

The second limitation relates to our experimental setting, which may have influenced the frequency of bee–flower interactions. As there were limited flowers in the cage, transmission could be more or less frequent here than in nature—that is, the bees could be visiting flowers more often in the cage in order to get as much food as possible, or they could be visiting less often if resources were drawn down quickly. Our estimates of transmission could therefore be higher or lower than they are in reality. However, the point of our experiment was not to estimate absolute rates of transmission but instead to experimentally test whether floral transmission of *A. micheneri* is possible via *O. lignaria* . Nonetheless, future studies could address this limitation by conducting experiments in larger, seminatural enclosures or open field conditions to better approximate natural bee–flower interactions.

## 5 Conclusion

Our study provides empirical support for the environmental transmission hypothesis, demonstrating that flowers are hubs of transmission for putatively beneficial bacteria. These results suggest that future research should test the persistence of microbial transmission across multiple generations and different environmental conditions. Our findings emphasize the role of environmental transmission as a mechanism for microbial acquisition in host‐associated symbionts.

## Author Contributions

**Magda Argueta‐Guzmán:** data curation (lead), formal analysis (lead), investigation (lead), methodology (lead), visualization (lead), writing – original draft (lead). **Marko J. Spasojevic:** project administration (equal), resources (lead), supervision (equal), writing – review and editing (equal). **Quinn S. McFrederick:** conceptualization (lead), data curation (equal), formal analysis (equal), funding acquisition (lead), investigation (equal), methodology (lead), supervision (lead), writing – review and editing (lead).

## Conflicts of Interest

The authors declare no conflicts of interest.

## Data Availability

DNA sequences from *A. micheneri* have been uploaded to the NCBI nucleotide database (SUB14727451).
